# Establishment of a Sandwich-ELISA for simultaneous quantification of bovine pregnancy-associated glycoprotein in serum and milk

**DOI:** 10.1371/journal.pone.0251414

**Published:** 2021-05-12

**Authors:** Tony Krebs, Isabel Kilic, Katja Mütze, Sonja Kleinhans, Daniel Lücking, Mark Hennies, Jens Tetens

**Affiliations:** 1 Department of Animal Sciences, Georg-August-University, Goettingen, Lower Saxony, Germany; 2 Hessischer Verband für Leistungs- und Qualitätsprüfungen in der Tierzucht e.V., Alsfeld, Hesse, Germany; 3 TECOdevelopment GmbH, Rheinbach, North Rhine-Westphalia, Germany; Xavier Bichat Medical School, INSERM-CNRS - Université Paris Diderot, FRANCE

## Abstract

Bovine pregnancy-associated glycoproteins (**boPAG**) are expressed by trophoblast cells in the bovine placenta. The multigene family of boPAG belongs to the group of aspartic proteases. The accumulation and circulation in maternal blood and milk has made boPAG very useful and important for pregnancy diagnosis in cattle. The goal of the present study was to develop and validate a new Sandwich-ELISA which allows the detection of boPAG in maternal serum and whole milk. Therefore, 984 serum and 928 milk samples were collected monthly from 231 Holstein Friesian cows (*Bos Taurus*) from one week after insemination (**p.i.**) until six weeks postpartum. The ELISA is able to identify a cow as being pregnant at day 30 p.i. in serum and at day 40 p.i in milk with threshold values of 1.0 ng/ml in serum and 0.0165 ng/ml in milk. The postpartum half-life of boPAG was estimated to be 6.4 days in serum and 7.1 days in milk. The boPAG profile established during pregnancy in serum and milk showed a typical pattern. The amount of boPAG found in milk was 1.5 % of the amount of boPAG present in serum. In conclusion, a Sandwich-ELISA has been developed to quantify boPAG in serum and in whole milk simultaneously with the same test procedure. This is time saving for farmers and more efficient for laboratories.

## Introduction

An accurate and timely pregnancy diagnosis is of considerable economic relevance in livestock management, especially in the cattle industry. Traditionally, pregnancy testing is done by manual or ultrasonographic examination per rectum or with the detection of progesterone as a non-pregnancy specific marker in serum or milk. In the last three decades, the identification and immunological detection of "pregnancy-associated glycoproteins" (**PAG**) has turned out to be an alternative method for pregnancy diagnosis [1–4].

These proteins are expressed by different cell types of the placenta. They are products of an unusual gene-family, which phylogenetically belongs to aspartic proteinases and is present in the Cetartiodactyla order [5]. PAG are particularly numerous in the Bovidae with their synepitheliochorial placenta [5, 6]. In cattle, roughly 20 different PAG members and related paralogs are known with largely varying temporal and spatial expression and glycosylation patterns during gestation [5, 7, 8]. Until now, the knowledge about the exact number and function of bovine PAG (**boPAG**) is incomplete [7], but the accumulation and circulation in maternal blood and milk have made boPAG a very useful and important tool for pregnancy diagnosis in cattle [5, 9–12]. Different studies demonstrated that boPAG can be used as reliable pregnancy markers in serum and milk, applicable as early as approximately day 25 post breeding [5, 13–15]. Thus, the detection of boPAG in serum or milk is an alternative method to transrectal ultrasonography or progesterone assays. Furthermore, boPAG-determination allows the direct identification of a placental product present in the maternal system that can be used as a marker of a viable pregnancy [16]. Some studies already figured out that boPAG are effective at identifying cows that will undergo early fetal mortality or late embryonic loss and are potential markers for a healthy placental function [15, 17, 18]. For these reasons, the measurement of boPAG in blood or milk is an important and powerful diagnostic tool for livestock management.

Today, there are different ELISAs available for detection of PAG in bovine milk and serum but most of the milk ELISA use skimmed milk instead of whole milk. In some studies, PAG has been measured in unskimmed milk, but using a commercial test kit, which cannot quantify PAG concentrations in milk [11, 12, 19].

The present study addresses the establishment and validation of a new ELISA that quantifies boPAG concentrations in blood or whole milk samples in one ELISA system within a few hours. This is time saving for farmers and more efficient for laboratories, since only one test is necessary which enables a parallel measurement of blood and milk samples.

## Materials and methods

The study is in accordance with the German legal and ethical requirements of appropriate animal procedures. Animals were not purposely euthanized for this study. Tissue samples were taken during the conventional slaughter process. The consultation of the institutional Animal Welfare Body is documented under no. E5-18.

### Tissue collection for protein purification

For protein purification, cotyledon samples from different pregnancy stages were collected from an abattoir located in Germany, afterbirth cotyledons were obtained from a local dairy farm directly after calving. Uteri of pregnant cows (*n* = 16) were opened approximately 20–30 min after killing. Thereafter, the cotyledons were dissected from the caruncula and extensively washed with 0.9 % NaCl. Subsequently, the samples were immediately stored on ice and transported to the laboratory where they were stored at -20°C until further processing. The gestation stage was estimated by measuring the crown-rump-length of the fetuses [20]. Overall, the collected cotyledons were divided into four different pregnancy stages: 35–90 days of gestation (early pregnancy), 91–180 days of gestation (mid pregnancy), 181–240 days of gestation (late pregnancy) and afterbirth samples.

### Protein extraction

The protein extraction was performed according to Zoli et al. [1] and Klisch et al. [21] with some modifications. Cotyledonary tissue was thawed, weighed and homogenized with an Ultra-Turrax (Ultra-Turrax T18 digital, IKA, Germany) in potassium phosphate buffer (0.01M KH_2_PO_4_, 0.1M KCl; pH 7.6) at 4°C. The buffer tissue ratio was 5:1 (v/w). Protease inhibitors (0.2 mM phenylmethylsulfonyl fluoride, 0.2% sodium EDTA) were added during the homogenization process. The mixture was stirred for 20 min at 4°C. Then the homogenate was centrifuged at 3,000 x *g* and 4°C for 30 min. The pellet was discarded. The supernatant was transferred in a beaker and stirred. Ammonium sulfate was slowly added to achieve 40% saturation. Thereafter the supernatant solution was gently stirred at 4°C for 1 h. Then it was centrifuged at 3,000 x *g* and 4°C for 30 min. Again, the pellet was discarded and the supernatant was adjusted to 80% ammonium sulfate saturation. After stirring the sample at 4°C for 1 h, it was centrifuged at 27,000 x *g* and 4°C for 1 h. The pellet was retained and dissolved in Tris/Cl buffer (0.01 M; pH 7.6). Following this step, the sample was stored at -20°C until further analysis.

### Chromatography

For protein purification, a fast protein liquid chromatography (**FPLC**) with the following steps was used: anion-exchange (Acetate basis), cation exchange (Acetate basis), gel filtration, cation exchange (Tris basis), hydrophobic interaction chromatography and anion exchange (Bis-Tris basis). Between the different FPLC steps the fractions were checked for boPAG content by using an available ELISA previously established by Friedrich and Holtz [9]. This ELISA was established on the basis of an existing RIA [22] and uses an anti-boPAG-1-IgG polyclonal rabbit antiserum for specific binding of PAG [9]. Prior to chromatography, samples were thawed and filtered through a syringe filter (0.22 μm; polyethersulfone, Carl Roth, Germany) to avoid clogging of the different columns. In the first step, the filtered samples were desalted in Tris/Cl buffer (0.01 M; pH 7.6) by passing it through a Sephadex G-25 column (HiTrap Desalting, 5 ml, GE Healthcare, USA). Following this, the samples were loaded to a Source 30Q (GE Healthcare, USA) packed column (20 ml, HiScale 16, GE Healthcare, USA), which was equilibrated with Tris/Cl buffer (0.01 M; pH 7.6). After unbound protein had washed through, elution of the loaded samples (40 ml) was performed using six steps of increasing ionic-strength buffer by adding NaCl (0.02 M, 0.04 M, 0.08 M, 0.16 M, 0.32 M and 1 M). The flow rate was 10 ml/min and the absorbance was measured at 280 nm. Fractions were collected automatically and were checked for boPAG content by ELISA [9]. The fractions with antigenic activity were pooled. The pools were submitted to buffer exchange using a HiTrap Desalting column equilibrated in ammonium acetate buffer (0.01 M; pH 5.2). The concentrated fractions were further purified on a Source 30S (GE Healthcare, USA) packed column (Tricorn 10/100, GE Healthcare, USA) equilibrated with the sample buffer. The loaded samples (90 ml) were eluted with the above-mentioned gradient of NaCl. The protein content was monitored by measuring the UV absorbance at 280 nm. Again, all fractions were analyzed by ELISA [9]. The boPAG-containing fractions were pooled and submitted to gel filtration on a Superdex 200 column (Superdex 200 Prep Grade in a XK 26/70 Column, GE Healthcare, USA), equilibrated in PBS (0.68 M NaCl, 0.0405 M Na_2_HPO_4_, 0.0075 M KH_2_PO_4,_ 0.0135 M KCl; pH 7.3) plus 0.5 M NaCl buffer. A maximum of 10 ml per sample was loaded on the column. Fractions of 5 ml were collected. The flow rate was 2 ml/min and the absorbance was recorded at 280 nm. In the next step, all boPAG-containing and pooled fractions were subjected to a buffer exchange to cation buffer (15 mM Na_2_HPO_4_, 15 mM HCOONa, 35 mM C_2_H_3_NaO_2_; pH 5.25) using the desalting column. Subsequently, a second cation exchange was carried out using the Source 30S packed column equilibrated with the same buffer. The bound proteins were eluted with the exponential gradient of NaCl. The protein content was monitored by measuring the UV absorbance at 280 nm. After cation exchange, ELISA-checked and pooled fractions were loaded onto a column (1 ml HiTrap Phenyl HP, GE Healthcare, USA) for hydrophobic interaction chromatography after the addition of the same volume of 4 M (NH_4_)_2_SO_4_. Previously, the columns had been equilibrated with a buffer containing 50 mM Na_2_HPO_4_ and 2 M (NH_4_)_2_SO_4_ (pH 7). Proteins were eluted using a linear gradient to water. Fractions of 0.5 ml were collected and assayed. Those with high antigenic activity were pooled, and buffer was exchanged to an anion exchange buffer (35 mM BisTris, 25 mM Tris; pH 9.0) using a fresh desalting column. Following this, a second anion exchange was performed using a Source 30Q packed column (Tricorn 10/100 equilibrated with anion exchange buffer). After elution of the unbound proteins, the exponential NaCl gradient was applied at a flow rate of 3 ml/min. 1 ml fractions were automatically collected and analyzed by ELISA [9]. The boPAG-containing fractions were pooled and equilibrated in PBS buffer using a desalting column. Afterwards, they were stored at -20°C until further processing.

### Polyclonal antibody production

Anti-boPAG antibodies were produced using seven boPAG-fractions of different pregnancy stages from chromatography. Immunizations of rabbits against PAG from early, mid, late pregnancy and afterbirth were performed by ImmunoGlobe Antikörpertechnik GmbH (Himmelstadt, Germany). In total seven rabbits were immunized with boPAG in PBS (early pregnancy (2 rabbits), mid pregnancy (1 rabbit), late pregnancy (3 rabbits) and afterbirth (1 rabbit)). Each rabbit received 5–7 times multiple intra dermal injections with approximately 250 μg purified boPAG-fraction with Montanide ISA 206 (Seppic, France) as adjuvant. Blood collection were carried out two and three weeks after each antigen injection starting after the third boost and final collection after the last boost. The antisera within rabbits were pooled.

Afterwards, each antibody was tested with itself and the other antibodies for coating and as biotinylated antibodies. In contrary to expectations, two different antibodies against late pregnancy PAG preparations (IgG 1438 and IgG 1440) were found to be most suitable for the development of the Sandwich-ELISA. This pair of antibodies showed the best differentiation between pregnant and non-pregnant animals in combination with high specific PAG binding and low background in the assay system.

### Sandwich-ELISA for detection of PAG in serum or milk

The PAG-Sandwich-ELISA utilizes 96 well microtiter plates (Costar 2592, Corning, USA). These plates were coated with 100 μl of anti-PAG polyclonal rabbit antibody (IgG 1438; raised against PAGs from late pregnancy) at a concentration of 1 μg/ml in coating buffer (0.05 M NaHCO_3_; pH 9.6). Antibodies were purified by using rmp Protein A Sepharose Fast Flow (GE Healthcare, USA). After overnight incubation at 4°C, the wells were blocked with washing buffer (10 % PBS, 0.05% Tween 20) and then washed five times with 350 μl washing buffer. Plates were stabilized by using 300 μl of 20% sucrose solution. After decantation, the plates were dried at room temperature and stored with silica gel at 4°C until use. A standard stock solution of 10 ng/ml was prepared from a cotyledonary extract (crude extract) from mid pregnancy in standard buffer (PBS-T (PBS with 0.05% Tween 20), 0.1 M NaH_2_HPO_4,_ 10% PAG-free bovine serum (from non-pregnant animals)) and stored at -20°C until use. The boPAG-content of the extract was determined with the same ELISA mentioned above [9]. For the preparation of a standard curve, the standard stock was diluted 1:20 in dilution buffer (PBS-T, 0.1M NaH_2_HPO_4_, 10 % PAG free-bovine serum). Afterwards, two-fold serial dilutions were prepared freshly before every use. This procedure resulted in seven standards with the following concentrations: 500 pg/ml, 250 pg/ml, 125 pg/ml, 62.5 pg/ml, 31.3 pg/ml, 15.6 pg/ml and 7.8 pg/ml. Dilution buffer was used as 0-Standard (0 pg/ml) and negative control. Positive control samples were prepared from two serum or milk samples with high and midrange boPAG-concentrations. For this purpose, the serum sample with a high concentration (33.5 ng/ml) was diluted 1:100 in dilution buffer and the serum sample with a medium concentration (3.5 ng/ml) was diluted 1:20. The milk sample with a high (442.6 pg/ml) and medium concentration (48.4 pg/ml) was used undiluted. The same control samples were used on each plate throughout the experiment.

Bovine serum samples were diluted in dilution buffer 1:10 in early pregnancy (<30 d post insemination (**p.i.**)), 1:100 in mid pregnancy (>30 d p.i.) and 1:1,000 in later pregnancy (>150 d p.i.). Milk samples were used undiluted in the beginning of gestation (<160 d p.i.) and diluted 1:10 until the end of gestation (>160 d p.i.). The dilutions at the aforementioned gestation stages were necessary to allow accurate concentration measurement of the samples in the range of the standard curve. All standards, samples and controls were assayed in duplicate.

For the first step, 50 μl of matrix solution (0.05% PBS-T, 0.1 M NaH_2_HPO_4_, 10% PAG-free bovine milk (from non-pregnant animals), 10% PAG free-bovine serum) were added into each well. Afterwards, 50 μl of prepared standard, control or pre-diluted sample were added, and the mix was incubated for 2 h in the dark and at room temperature (20–25°C) on a shaker (500 rpm). After incubation, the plate was washed three times with 350 μl of diluted washing buffer before adding biotin-conjugated anti-boPAG polyclonal rabbit antiserum (35 ng/ml) to the wells. The antibody (IgG 1440; raised against PAGs from late pregnancy, Protein A purified) was conjugated to Biotinamidohexanoic acid N-hydroxysuccinimide ester (**biotin**). The biotin antibody conjugate was diluted in biotinylated antibody buffer (0.136 M NaCl, 0.02 M Na_2_HPO_4_, 0.01 M EDTA, 0.005% chlorhexidine digluconate, 0.1% gelatine, 0.05% Tween 20, 1% BSA, 10% normal rabbit serum, 50% PAG free- bovine milk). To each well, 100 μl of diluted biotin antibody conjugate was added, followed by 30 min incubation in the dark at room temperature on a shaker (500 rpm). After three washing steps with 350 μl of diluted washing buffer, 100 μl of streptavidin conjugated to horseradish peroxidase (EC 1.11.1.7) (**HRP**) was added into each well. The streptavidin-HRP conjugate was diluted 1:500 in HRP buffer (0.136 M NaCl, 0.02 M Na_2_HPO_4_, 0.01 M EDTA, 0.005% chlorhexidine digluconate, 0.1% gelatine, 0.05% Tween 20, 1% BSA, 10% normal rabbit serum). The plate was incubated for 30 min in the dark at room temperature on a shaker (500 rpm), followed by 5 additional washes with 350 μl of diluted washing buffer. Then 100 μl of 3,3′,5,5′-Tetramethylbenzidin (**TMB**) substrate (TMBS, SurModics, MN, USA) were added, followed by an incubation for 20 min in the dark at room temperature on a shaker (500 rpm). The enzyme reaction was stopped by the addition of 100 μl of 1 M HCl. The color changed from blue to yellow and the color intensity was measured spectrophotometrically at 450 nm with a 650 nm reference filter using an EMax Plus Microplate reader (Molecular Devices, USA) with software SoftMax Pro 6.5.1 (Molecular Devices, USA). Automatic data reduction was done using a 4-parameter logistic (4-PL) curve fit.

For development and validation, the new boPAG Sandwich-ELISA was standardized with boPAG-1 which was used in an well-established double-antibody ELISA in our laboratory [9]. During assay development, 37 serum samples were measured in both assays. The results are shown in the S1 Fig of the supplementary material. For further validation, all collected blood samples in this study were analyzed on both ELISA systems. The measurement of milk samples with the double-antibody ELISA was not possible, as this ELISA is not sensitive enough to analyze boPAG-content in milk.

In order to determine the intraassay variability of the new Sandwich-ELISA, four serum (515 pg/ml; 155 pg/ml; 65 pg/ml; 46 pg/ml) and four milk samples (87 pg/ml; 51 pg/ml; 44 pg/ml; 17 pg/ml), were analyzed 10 times in duplicate within one plate in three independent assays. On the other hand, the interassay variability was determined measuring two milk and serum samples in duplicate on 20 plates. These samples are the same as those used as positive controls. The detection limit was determined measuring 20 0-Standard samples in duplicate plus three standard deviations. A total of 52 assays were performed for the analysis of all samples in the validation study (26 serum assays and 26 milk assays).

### Serum and milk collection

For the validation of the assay and the establishment of PAG profiles, 984 blood and 928 milk samples were collected monthly from 231 Holstein Friesian cows (*Bos Taurus*) from one week after insemination (**p.i.**) until six weeks postpartum (**p.p.**). The last calving of the sampled cows was a minimum of 87 days ago. The animals were housed on different farms in Lower Saxony and Hesse (Germany). The blood sample and the corresponding milk sample for each animal were collected on the same day. Confirmation of pregnancy in these animals was carried out by regular analysis of all serum samples using the well-established ELISA by Friedrich and Holtz [9]. Furthermore, the pregnancy was verified by the birth of a healthy calf. This information was provided directly from the farmers. Cattle were considered non-pregnant if the date of the last insemination was at least 60 days in the past and the pregnancy tests performed until then had shown a negative result. These animals continued to be sampled at monthly intervals until a new insemination was performed by the farmer. Samples from animals with an abort, animals that had no more than one sample or animals which have left the farms for different reasons (e.g. udder diseases, unsuccessful rebreeding etc.) were not included in the further analysis. In total 5 animals had an abortion (between days 96 and 163 after insemination). Three of them had lower PAG concentrations in serum and milk than animals that carried their calves to term. One animal showed higher PAG concentrations in serum and normal concentrations in milk compared to the animals that calved at term and the last animal showed no differences in serum and milk concentrations in comparison to animals with a normal pregnancy. Similar results are described in literature [17, 18, 23]. We excluded these animals from the analysis due to the small sample size. Milk samples were not examined in the dry period.

All blood samples in this study were collected from tail blood vessels. Approximately 12 ml of whole blood was captured in sample tubes for serum collection with separating agent (KABE LABORTECHNIK GmbH, Germany). They were immediately cooled and shipped to our laboratory. Then the blood was centrifuged at 2,800 x *g* for 10 min at 4°C. The serum was stored at -20°C until further processing. Milk samples (approximately 8 ml from each cow) were stripped from a healthy quarter before milking and stored in milk preservation tubes with ProClin as preservative (KABE LABORTECHNIK GmbH, Germany) at -20°C until assayed. All serum and milk samples were vortexed following thawing and before dilution or analysis steps.

### Statistical analysis

All experimental results were analyzed with R 3.6.1 (R Development Core Team, Austria). A nonlinear regression (for all samples) and a linear regression (for samples from gestation day 30 onwards) were used to estimate the correlation between boPAG-concentration and days after insemination. To determine the earliest gestation day at which the test can significantly differentiate between pregnant and nonpregnant animals, a one-sided ANOVA was used. Post hoc evaluation was performed with a two-sided Dunnett T-Test [24]. The results were considered significant at *P* < 0.05.

The clinical sensitivity, clinical specificity, the positive predictive value (**PPV**) and the negative predictive value (**NPV**) were calculated for various threshold values. Therefore, the R-package “pROC” [25] was used. All blood and milk samples collected throughout gestation were included in this analysis. The clinical or diagnostic sensitivity indicates the probability of how well the ELISA correctly identifies a pregnant cow as pregnant, whereas the clinical or diagnostic specificity indicates the probability of how well the ELISA correctly identifies a cow as open. These terms should not be mistaken with the analytical sensitivity or specificity of a test system. Since the diagnostic sensitivity and specificity do not give any information about the probability of the test giving the correct diagnosis in the population tested, we calculated the positive predictive value and the negative predictive value. The positive predictive value of the test system is the likelihood that a cow with a positive test result actually is pregnant. The negative predictive value is the opposite of the positive predictive value. It is the likelihood that a cow with a negative test result is not pregnant.

A receiver operating characteristic (**ROC**) analysis was done [26] to determine the optimal cutoff value for serum and milk pregnancy testing. Samples were taken as experimental units in this analysis. The Youden’s index was used to find the best cutoff value that optimizes sensitivity and specificity. It is the threshold, that maximizes the distance to the identity line [27]. Furthermore, the area under curve (**AUC**) was calculated to measure the ability of the test to correctly classify pregnant and nonpregnant cows. A perfect test has an AUC of 1.0.

## Results

The standard curve of the Sandwich-boPAG-ELISA showed a linear pattern. The characteristics of the ELISA are shown in Table 1. As expected, the highest variation was observed in the control sample with the lowest concentration. The correlation of the serum concentrations between the Sandwich-boPAG-ELISA and the established ELISA in our lab was r = 0.96 (*P*<0.001), which is a clear indication that boPAG (and therefore pregnancy) recognition of both assay types is comparable. Recovery of serial dilution of serum and milk samples was 109.5% and 112.1%, respectively.

**Table 1:** Properties of the Sandwich-ELISA for measuring pregnancy-associated glycoprotein in serum and milk.

	Serum	Milk
Sample volume (μl)	50	50
Measuring range (pg/ml)	78–5,000	7.8–500
Detection limit (pg/ml)	7.43	0.74
Dilution linearity (%)	109.5	112.1
Mean recovery rates (%)	99.5	100.5
Intraassay CV (%)	1.9	2.5
Interassay CV (%)		
3.5 ng/ml	4.6	
33.5 ng/ml	6.1	
48.4 pg/ml		11.4
442.6 pg/ml		7.0

### Serum analysis

**Fig 1:**
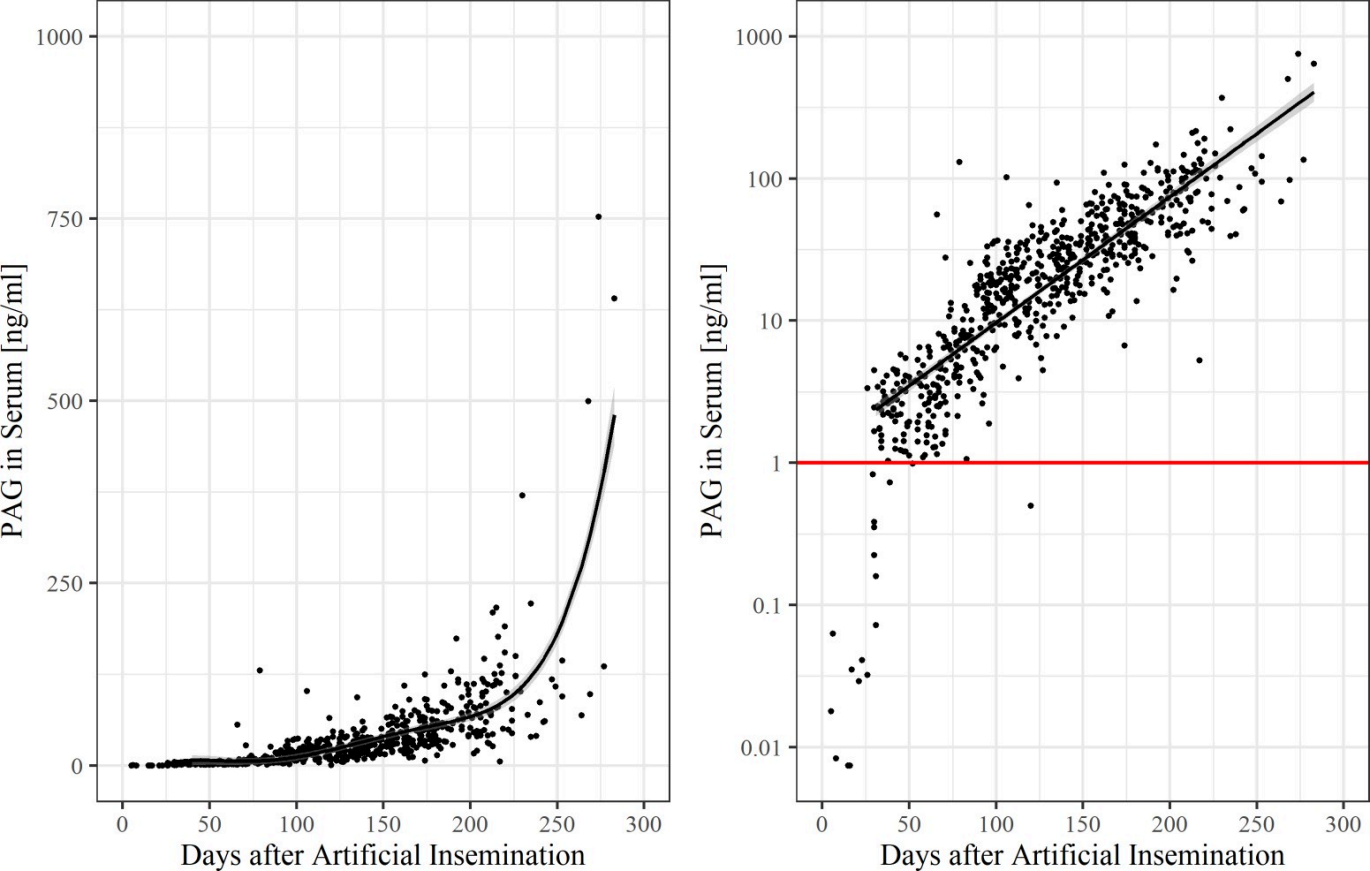
Pregnancy-associated glycoprotein concentrations during pregnancy in serum. The correlation between pregnancy-associated glycoprotein concentration and days after insemination was estimated with a non-linear regression for all samples (R^2^ = 0.61, *P*<0.001) (left) and with a linear regression for samples from gestation day 30 onwards (R^2^ = 0.74, *P*<0.001) (right). The red line indicates the threshold value. Note the log-scale of the y-axis in the right figure.

155 serum samples were collected from 62 nonpregnant animals and 666 serum samples were collected from 154 pregnant animals monthly throughout pregnancy until six weeks postpartum. These were the samples that met requirements for further analysis. Unless otherwise stated, results in the following section are presented as 10-day-means ± SEM. Individual boPAG-concentrations in the course of pregnancy are shown in Fig 1. BoPAG was detectable in serum before day 30 p.i., but only in very low concentrations. The serum boPAG concentration began to rise in maternal blood around day 30 p.i. reaching an average concentration of 2 ng/ml ± 0.2 ng/ml by day 40 p.i. and then were stable until day 70 p.i.. Afterwards, the circulating boPAG rose steadily through the rest of pregnancy. Approximately at day 250 p.i., the average serum concentration of boPAG declined to 86.7 ng/ml ± 15.4 ng/ml. Thereafter, the serum boPAG concentrations rose until parturition.

**Fig 2:**
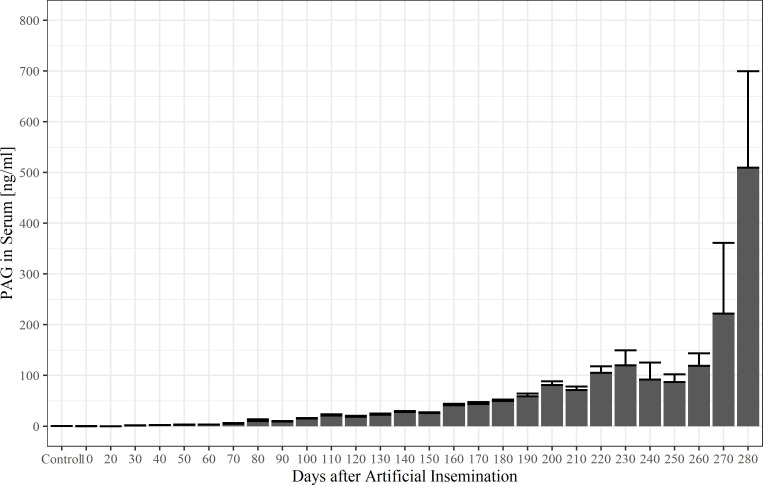
Serum pregnancy-associated glycoprotein concentrations (10-day-means ± SEM) in pregnant and non-pregnant control cows (control).

The overall mean boPAG concentration of non-pregnant cows was 0.12 ng/ml ± 0.03 ng/ml and the overall mean boPAG concentration of pregnant cows was 34.1 ng/ml ± 2.1 ng/ml. 10-day means of boPAG serum concentration throughout pregnancy in comparison to the nonpregnant control group are shown in Fig 2. Average boPAG-values for pregnant cows were significantly different from those of the nonpregnant control group from day 100 onwards (*P* = 0.002, Dunnett-Test). This is the result of a considerably high variation in the concentrations of boPAG in serum, as indicated by the standard errors from the average values.

The results of the ROC analysis at various threshold values are shown in Table 2. The corresponding ROC curve (S2 Fig) and confusion matrices (S1–S5 Tables) can be found in the supplemental material. Across all serum samples, this approach resulted in an optimal threshold value of 1.0 ng/ml with an AUC of 0.988, a sensitivity of 97.1%, and a specificity of 95.5 %. The PPV is 98.9% and the NPV is 88.6%. A serum sample <1.0 ng/ml would identify a cow as open, whereas a sample with a serum boPAG concentration >1.0 ng/ml would identify a cow as being pregnant. On average, this value was reached at day 30 p.i. by a pregnant cow.

**Table 2:** Accuracy of a pregnancy diagnosis based on the serum and milk pregnancy-associated glycoprotein level assessed with the newly established ELISA at various threshold values.

	Serum					Milk				
Threshold (ng/ml)	0.4	1.0	1.5	2.0	2.5	0.01	0.0165	0.02	0.025	0.16
Sensitivity (%) (no./no.)	97.7 (651/666)	97.1 (647/666)	94.0 (626/666)	91.1 (607/666)	88.4 (589/666)	97.3 (616/633)	95.3 (603/633)	93.4 (591/633)	90.8 (575/633)	56.2 (356/633)
Specificity (%) (no./no.)	89.0 (138/155)	95.5 (148/155)	98.7 (153/155)	99.4 (154/155)	99.4 (154/155)	85.2 (121/142)	91.5 (130/142)	93.0 (132/142)	95.1 (135/142)	98.6 (140/142)
PPV (%) (no./no.)	97.5 (651/668)	98.9 (647/654)	99.7 (626/628)	99.8 (607/608)	99.8 (589/590)	96.7 (616/637)	98.0 (603/615)	98.3 (591/601)	98.8 (575/582)	99.4 (356/358)
NPV (%) (no./no.)	90.2 (138/153)	88.6 (148/167)	79.3 (153/193)	72.3 (154/213)	66.7 (154/231)	87.7 (121/138)	81.3 (130/160)	75.9 (132/174)	69.9 (135/193)	33.6 (140/417)
Accuracy (%) (no./no.)	96.1 (789/821)	96.8 (795/821)	94.9 (779/821)	92.7 (761/821)	90.5 (743/821)	95.1 (737/775)	94.6 (733/775)	93.3 (723/775)	91.6 (710/775)	64.0 (496/775)

The numbers within the parentheses indicate the number of samples.

**Fig 3:**
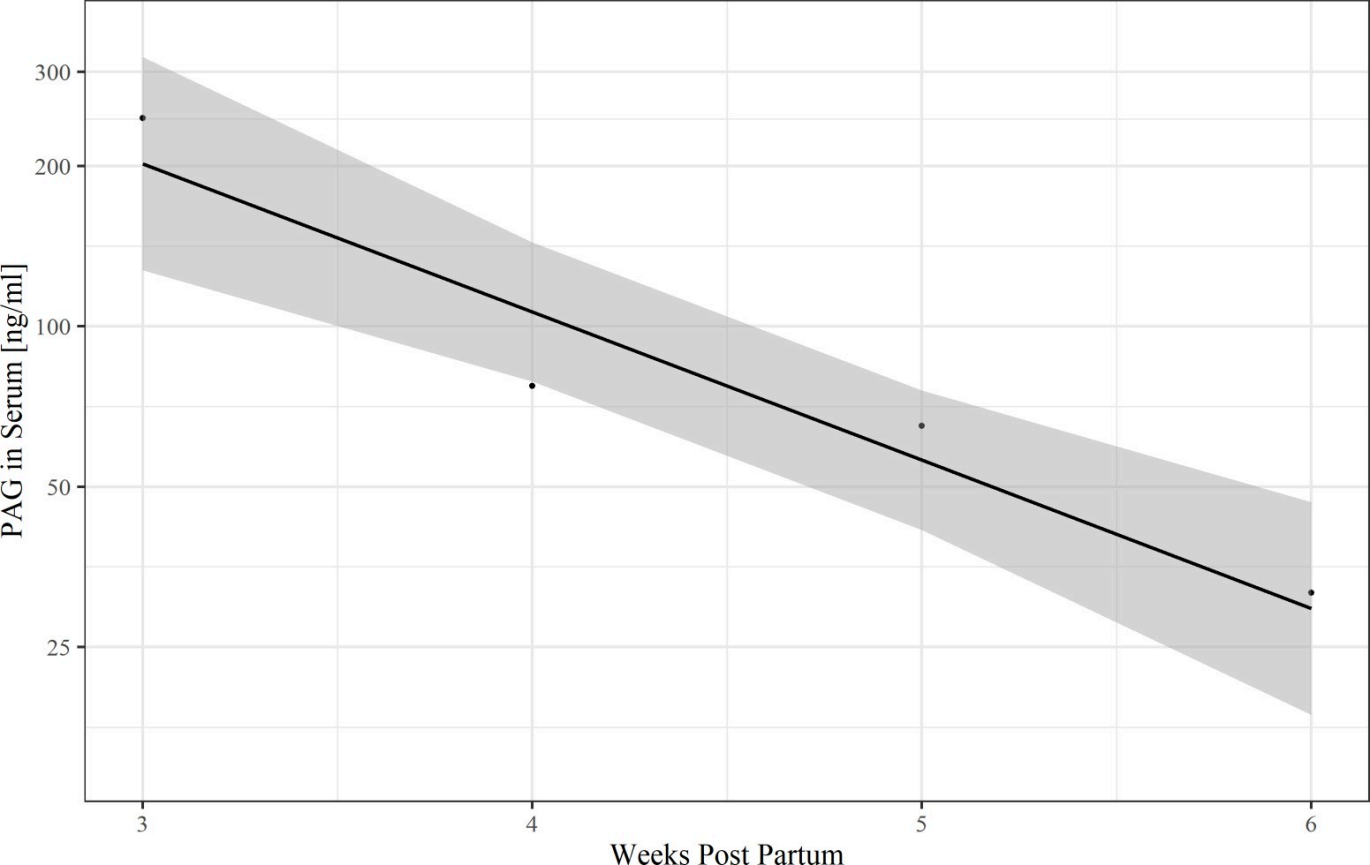
A ln-PAG-time graph to estimate PAG half-life in the postpartum period and to estimate the time at which PAG became undetectable (R^2^ = 0.90, *P* = 0.03).

Post-partum samples were obtained from eight animals (total number of samples *n* = 9) in the period from three weeks after calving to six weeks after calving. Except for one cow, all animals were sampled only once. In total, there were four samples from the third week p.p. (days 17; 20; 20; 21), one sample from the fourth week p.p. (day 25), one sample from the fifth week p.p. (day 35) and three samples from the sixth week p.p. (days 37; 39; 40). The mean boPAG concentration in the post-partum period is 135.7 ± 38.4 ng/ml. The average concentration of PAG in serum decreases from 246.1 ± 36.9 ng/ml in week three after parturition to 31.6 ± 7.2 ng/ml by post-partum week six. A simple linear regression model was fitted to the data after ln transformation (Fig 3) to estimate boPAG half-life in the post-partum period and to estimate the time point when boPAG become undetectable for the ELISA. The estimated post-partum half-life of serum boPAG was 6.4 days and boPAG was estimated to be undetectable for the ELISA three months postpartum given a first-order process of boPAG elimination.

### Milk analysis

633 milk samples were collected from 154 pregnant animals and 142 milk samples were collected from 62 non-pregnant animals that were also chosen for the serum sampling. Milk samples were not examined in the dry period from day 250 p.i. until calving. Unless otherwise stated, results in the following section are presented as 10-day-means ± SEM.

**Fig 4:**
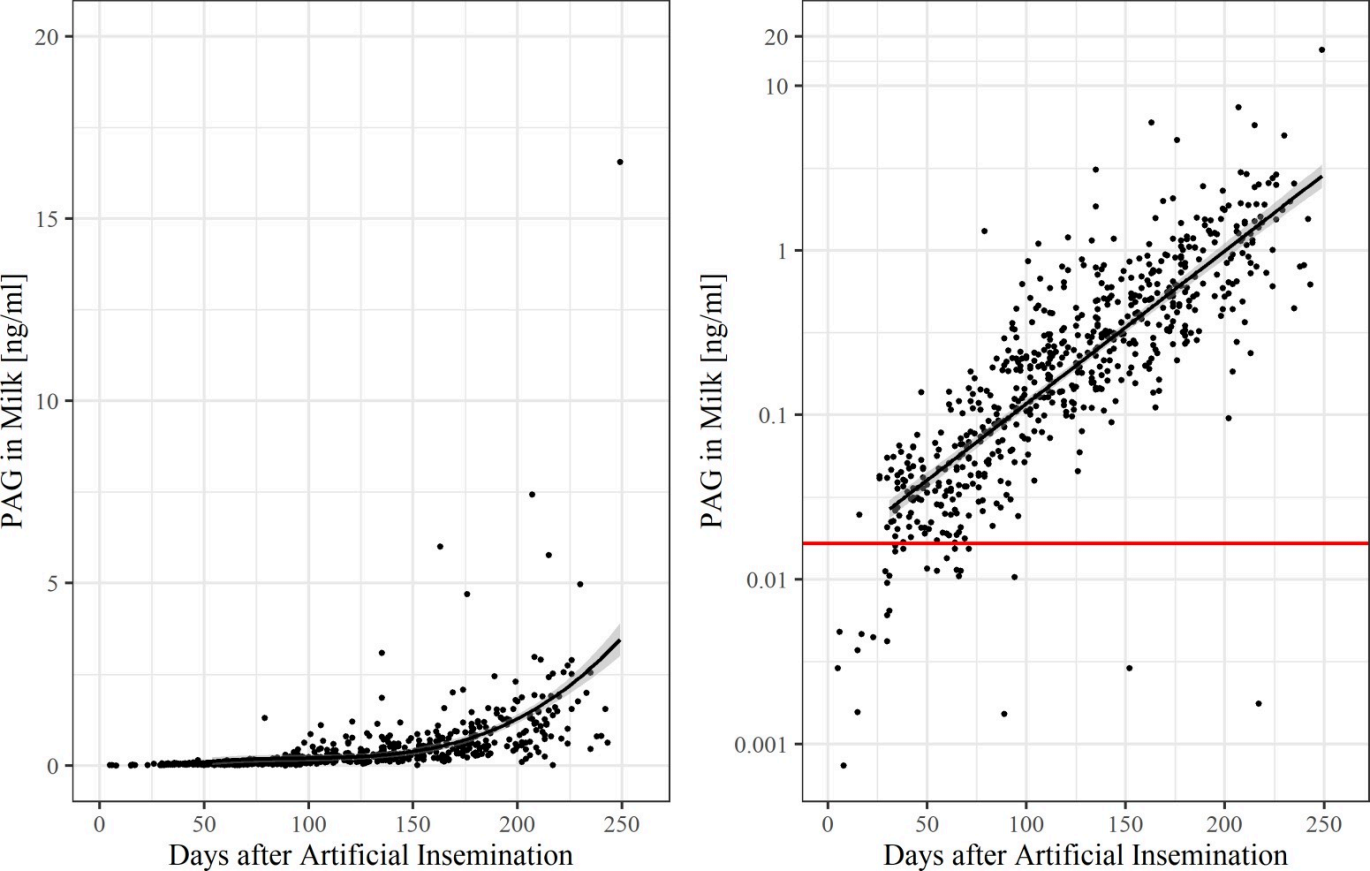
Pregnancy-associated glycoprotein concentrations during pregnancy in milk. The correlation between pregnancy-associated glycoprotein concentration and days after insemination was estimated with a non-linear regression for all samples (R^2^ = 0.34, *P*<0.001) (left) and with a linear regression for samples from gestation day 30 onwards (R^2^ = 0.67, *P*<0.001) (right). The red line indicates the threshold value. Note the log-scale of the y-axis in the right figure.

**Fig 5:**
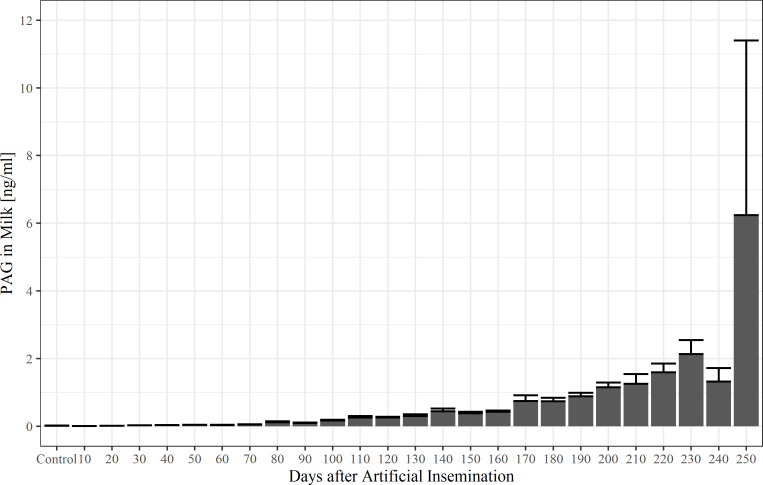
Milk pregnancy-associated glycoprotein concentrations (10-day-means ± SEM) in pregnant and nonpregnant control cows (control).

Individual concentrations of boPAG in milk during pregnancy are shown in Fig 4. BoPAG were detectable in milk before day 30 p.i., but only in very low concentrations. The boPAG concentration in milk began to rise at day 20 p.i. and reached a stable concentration of 0.025 ng/ml ± 0.006 ng/ml around day 30 p.i.. From day 60 p.i. until day 190 p.i., we found a slight increase in milk boPAG concentration up to 0.88 ng/ml ± 0.11 ng/ml. After 200 d p.i., the circulating boPAG rose steadily thought the rest of pregnancy. Nonpregnant animals had a mean boPAG concentration of 0.014 ± 0.006 ng/ml, whereas pregnant animals had a mean concentration of 0.47 ± 0.04 ng/ml. 10-day means of boPAG milk concentration in comparison to the nonpregnant control group is shown in Fig 5. Average boPAG values for pregnant cows were different from those of the nonpregnant control group from day 140 p.i. onwards (*P* = 0.008, Dunnett-Test).

The results of the ROC analysis for milk samples at various threshold values are shown in Table 2. The corresponding ROC curve (S3 Fig) and confusion matrices (S6–S10 Tables) can be found in the supplemental material. On the scale of all milk samples, this approach resulted in an optimal cut-off value of 0.0165 ng/ml with an AUC of 0.969, a sensitivity of 95.3%, and a specificity of 91.5%. The PPV is 98.0% and the NPV is 81.3%. A milk sample <0.0165 ng/ml would identify a cow as open, whereas a sample with a milk boPAG concentration >0.0165 ng/ml would identify a cow as being pregnant. On average, this value is reached at day 40 p.i. by a pregnant cow.

**Fig 6:**
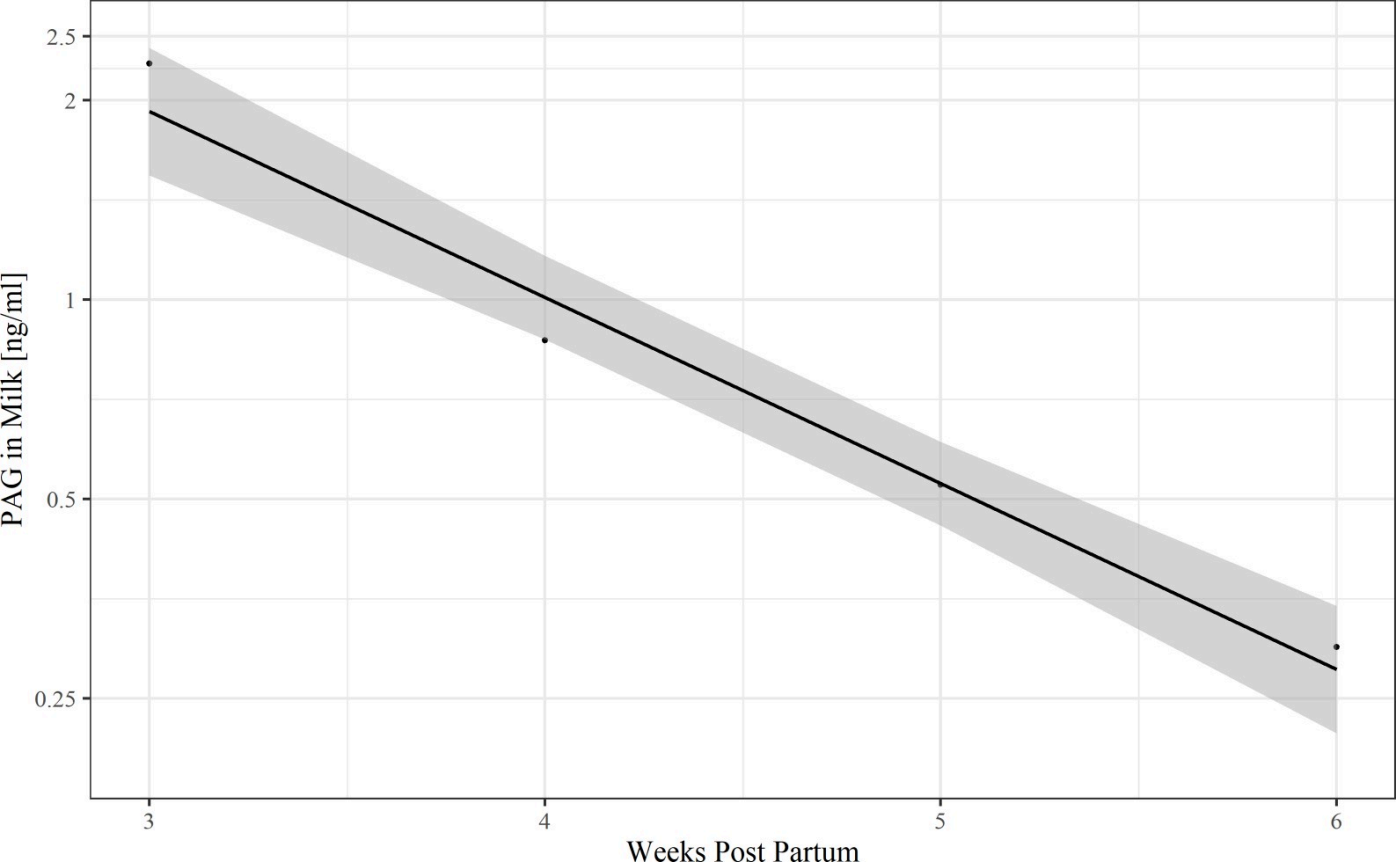
A ln-PAG-time graph to estimate PAG half-life in the postpartum period and to estimate the time at which PAG became undetectable (R^2^ = 0.97, *P* = 0.008).

In the post-partum period (three weeks p.p. to six weeks p.p.), the mean milk boPAG-concentration was 1.39 ± 0.41 ng/ml. The samples were collected from the same animals as described in the serum part. The mean milk boPAG concentration declined from 2.27 ± 0.5 ng/ml three weeks p.p. to 0.3 ± 0.06 ng/ml six weeks p.p. A simple linear regression model with ln-transformed boPAG as dependent variable was used (Fig 6) to estimate boPAG half-life in the post-partum period and to estimate the time at which boPAG becomes undetectable for the ELISA. Based on the estimated coefficient, the half-life of milk boPAG is 7.1 days. The time where milk boPAG become undetectable for the ELISA was estimated by linear regression to be two months postpartum given a first order elimination pattern.

### Correlation of serum and milk concentrations

**Fig 7:**
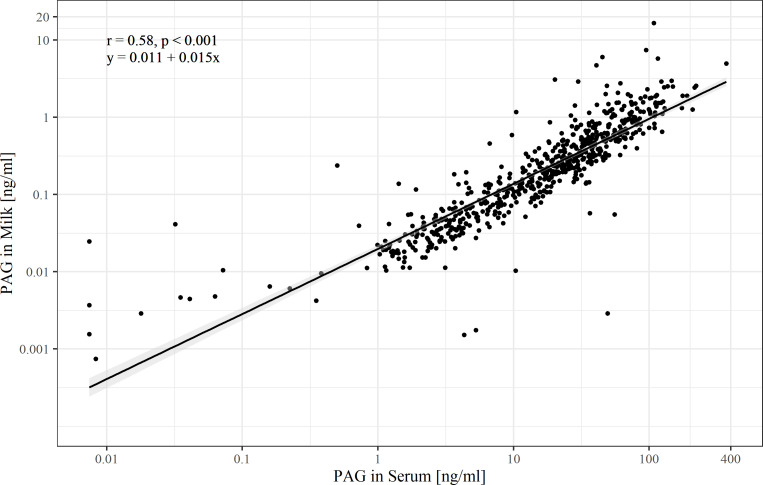
Comparison of concentrations of pregnancy-associated glycoproteins between plasma and milk of pregnant animals. Note the log-scale of both axes.

BoPAG-concentrations in milk amounted to 1.5% in pregnant animals and 11.7% in nonpregnant animals of that measured in serum. The correlation between milk and serum boPAG values was r = 0.58 (*P*<0.001) in pregnant and r = 0.11 (*P* = 0.17) in nonpregnant cows (Fig 7).

The rise of boPAG concentrations through the time of pregnancy is much sharper in serum compared to that in milk. Furthermore, the slope of the regression line reflects the greater concentrations of boPAG in serum compared with whole milk.

## Discussion

In cattle, an early and reliable pregnancy diagnosis is of considerable economic interest. ELISA based on the measurement of pregnancy-associated glycoproteins in serum or in milk offer an early possibility (28 days after insemination) to realize this intent. The detection of pregnancy specific proteins in serum or in milk in bovine species is a well-established diagnostic method [2, 9–11, 22, 28]. Pregnancy-associated glycoproteins are detectable in the maternal serum and in the milk throughout gestation and, therefore, are advantageous over progesterone testing. Progesterone testing has a prevalence for false positive results and the date of mating or artificial insemination is essential for correct pregnancy diagnosis [14, 22]. Ultrasonography is another valuable tool for detection of pregnancies as early as 26 days of gestation [14, 29]. However, the accuracy is limited under field conditions before day 30 of gestation and the pregnancy status is only guaranteed at the time of screening [14, 30]. Furthermore, expensive equipment and technical skills or a trained person are required when performing the ultrasound procedure [13, 29]. Regarding these facts, PAG assays are an alternative, reliable method for diagnosing pregnancies in cattle. Additional benefits of a PAG assay using milk are the avoidance of stressful effects during sampling (e.g. of venepuncture) and the omission of special equipment or experience.

Therefore, the goal of the present work was the development of an ELISA that is able to quantify boPAG concentrations in serum and milk simultaneously. To the best of our knowledge, only a few assays are described in literature, which are able to quantify PAG in bovine milk [9, 31, 32]. There is only one commercially available ELISA for detection of PAG in bovine milk [11, 12, 33], which is designed as a qualitative test and does not determine actual PAG concentrations. Instead, the results are classified as "not pregnant", "recheck" or "pregnant" [11, 12], based on the subtrahend (S-N value) from the optical density of the sample (S) and a negative control (N).

For our newly developed Sandwich-ELISA we decided to use polyclonal instead of monoclonal antibodies, as they have several advantages for our particular application. In cattle, roughly 20 different PAG members and related paralogs are known with largely varying temporal and spatial expression and glycosylation patterns during gestation [5, 7, 8]. Therefore, it is advantageous to use a broad spectrum of different antibodies to different epitopes, to ensure a reliable pregnancy diagnosis throughout pregnancy. Furthermore, the detection of multiple epitopes offers better sensitivity for detecting proteins that are present in low concentrations in a sample, which is the case for boPAG in milk and polyclonal antisera are less prone to posttranslational modifications of native proteins [34].

The validation of our newly developed boPAG-Sandwich-ELISA was performed on the basis of serum and milk samples from 216 cows throughout gestation. The earliest possible detection of boPAG in serum with our Sandwich-ELISA turned out to be day 17 p.i.. At this time point the concentration of boPAG in serum is very low (35 pg/ml). Such low concentrations of boPAG in early pregnancy should be looked at with caution. Zoli et al. [22] supposed that boPAG is formed in extraplacental tissues and that threshold values for a pregnancy diagnosis should take background or nonspecific levels (<0.5 ng/ml) into account. Therefore, results before day 28 p.i. should be interpreted with caution [22]. In the present study, the optimal threshold value for pregnancy testing in serum with the best sensitivity and specify after ROC analysis turned out to be 1.0 ng/ml. This cut-off value is higher than the value described by Zoli et al. (0.5 ng/ml) [22], almost the same as described by Green et al. (0.922 ng/ml) [10], and lower than the value described by Friedrich and Holtz (2ng/ml) [9] and is reached on average around day 30 p.i.. The differences in cut-off values do arise from the use of different antisera against boPAG. Due to the high variability of the PAG in bovids, the antisera thus probably detect different kinds of epitopes. The overall sensitivity and specificity of the test is quite similar to those determined by others using boPAG serum assays [9, 22, 35, 36]. The PAG profile obtained in the presented Sandwich-ELISA shows a nearly equal pattern as observed by others [2, 10, 32, 37].

In our study, we found a linear decline in ln-serum boPAG concentration in the postpartum period. Following a first-order elimination pattern, the postpartum serum half-life of boPAG was estimated to be 6.4 days. These findings are in line with those reported by other working groups with described half-lives ranging from 4.3 days to 8.86 days [5, 10, 22, 38]. Pohler et al. [18] and Wallace et al. [5] put the differences of serum half-life down to the detection of different members of the boPAG family or the detection of differentially glycosylated variants of the same boPAG family instead of a change in half-life during the course of pregnancy. With our assay, we estimated that PAG in serum were detectable until 90 days postpartum. Therefore, it is no problem for farmers to breed cows again after 50–70 days in milk. The assay can be used from the fourth week after insemination onwards. Thus, PAG from an earlier pregnancy are not an issue for the test at this point of time.

Studies about PAG-concentration in milk of cattle throughout pregnancy are rare in literature. There are only a few assays described, which quantify boPAG in milk [9, 31, 32, 39]. All those assays used skimmed milk instead of whole milk. In some studies, PAG has been measured in unskimmed milk, but using a commercial test kit, which cannot quantify PAG concentrations in milk [11, 12, 19].

With aid of our newly developed Sandwich-ELISA boPAG-detection in whole milk is possible as early as day 26 p.i. At this time point, the concentration of boPAG in milk is 41.2 pg/ml. This is an earlier time point as described by Friedrich and Holtz [9], who detected boPAG in milk at day 60 p.i. and as described by Gajewski et al. [32], who could not detect boPAG in milk during the first 3 weeks of pregnancy. In the present study, the optimal threshold value for pregnancy testing in milk turned out to be 16.5 pg/ml. On average, this value was exceeded around day 40 p.i.. This value is much lower than that reported by Gajewski et al., [32] who proposed a value of 0.2 ng/ml. Metelo et al. [31] and Friedrich and Holtz [9] did not specify threshold values, because those concentrations cannot be used as reference for confirming pregnancies. González et al. [40] proposed a cut-off value of 1.6 ng/ml for pregnancy testing in goat milk. The test can be used as early as day 32 p.i.. However, PAG concentrations in goat milk are ten times higher than for cows [40] The overall sensitivity and specificity of the Sandwich-ELISA using milk was high with 95.3 % and 91.4 % respectively. As a result, the PPV in our experiment was high (96.8 %) compared with the NPV (87.6 %). The overall accuracy of the test was 94.3 %. These results agree with others who have conducted milk assays [11, 19, 32, 35, 39]. Leblanc [12] reported a very high sensitivity (99.2 %) and specificity (95.5 %) of pregnancy diagnosis with the commercial milk test, but he used the test for confirmation of pregnancy after day 60 p.i. in cows previously diagnosed as pregnant by rectal palpation. Therefore, the number of nonpregnant cows depends on the accuracy of the rectal palpation [12].

The boPAG profile obtained in the Sandwich-ELISA shows lower concentrations in milk compared with other assays used for boPAG quantification in milk [9, 31, 32, 39]. The obtained average boPAG levels rose from 23.6 pg/ml ± 6.1 pg/ml on day 30 p.i. to 381.6 pg/ml ± 42.1 pg/ml on day 150 p.i. After day 150 p.i., a faster increase of boPAG levels was detectable until day 250 of pregnancy with an average concentration of 6.2 ng/ml. About day 200 p.i., the average concentration of PAG in milk exceeded 1.0 ng/ml. The average concentration of nonpregnant animals was 14.2 pg/ml ± 6.5 pg/ml. Friedrich [41] found a mean PAG-concentration of nonpregnant animals of 0.46 ± 0.10 ng/ml. In pregnant animals he described an average concentration of 0.67 ng/ml until day 100 p.i, a concentration of 0.83 ng/ml between day 100 p.i. and day 150 p.i. and a concentration of 1.28 ng/ml between day 150 p.i. and day 200 p.i. [41]. Before day 60 p.i., he could not determine concentrations above 1.0 ng/ml with the exception of two animals. In his study, he detected a rise in concentration at day 150 p.i., which is a similar date as found in our study [41]. Gajewski et al. [32] obtained a boPAG milk profile varying from 0.06 ng/ml in the 6^th^ week of pregnancy, through 0.20 ng/ml on average on day 119 of pregnancy, 1.28 ng/ml on day 168 p.i., to 4.84 ng/ml on day 201 p.i.. Furthermore, in their study they found a similar surge of boPAG concentration in milk around day 150 of pregnancy. Overall, we can conclude from these results, that our test found nearly the same pattern of boPAG in milk but lower concentrations. This may be due to the fact, that we used whole milk instead of skimmed milk. Since PAG are water soluble and associated with the aqueous portion of the milk, fat in whole milk may act as a source of interference [9, 19, 40]. Nevertheless, our newly developed Sandwich-ELISA is sensitive enough to be a useful tool for pregnancy diagnosis in whole milk as early as day 40 p.i..

In our study, we found a linear decline in ln-milk PAG concentration in the postpartum period as already described for serum samples. Following a first-order elimination pattern, the postpartum half-life of PAG in milk was estimated to be 7.1 days, which is quite similar to the half-life found in serum and it seems that there is no difference in the elimination rate of boPAG in serum and milk. However, the only difference is the lower concentration of boPAG in milk compared to serum which makes them undetectable for other assays around day 30 postpartum [41, 42]. With our assay, we estimated that PAG were detectable until 60 days postpartum.

In a last step, we compared the serum and milk PAG concentration throughout pregnancy. We found a correlation of r = 0.58 (*P*<0.001). The amount of PAG found in milk was 1.5 % of the amount of PAG present in serum, but the profiles were nearly parallel with exception of an aberration in late pregnancy. In literature information about the correlation between milk and blood PAG concentration is manifold ranging from 0.64 (whole milk and plasma of Holstein cows from 25 to 102 d in gestation [11]), 0.70 (skimmed milk and plasma of Holstein cows from 25 to 220 d in gestation [32]) and 0.79 (whole milk and plasma of Holstein cows from 23 d in gestation; [19]) to 0.81 (skimmed milk and serum of Holstein cows from day 51 to 250 in gestation [9]). Reasons for the relatively low correlation coefficient of our study in comparisons to the findings of the other studies are the individual variability of the boPAG concentration and the use of the more complex matrix whole milk instead of skimmed milk.

There are also different information in literature about the amount of PAG in milk compared to blood ranging from 0.6 % - 16.7% in quantitative assays [9, 31, 32, 39] and 50 % in the commercial test kit [11, 19]. To some extent the amount fluctuates along the course of pregnancy [32, 41]. The reasons for those phenomena are multifactorial. BoPAG levels are negatively correlated with increased milk production [11]. Furthermore the concentration of circulating PAG in blood or in milk is also influenced by other factors such as breed, body weight, parity status of the dam, foetal sex, foetal number and foetal birth weight [5]. The transport mechanisms of PAG from plasma across the mammary gland into milk are not fully understood until now. There are roughly 20 different boPAG members and related paralogs with a huge range in their temporal and spatial expression and glycosylation patterns during gestation [5, 7, 8]. Furthermore, it should be kept in mind, that every assay detects different members of the PAG family or differentially glycosylated variants of the same PAG with the result of often perplexing differences in studies [5]. Probably there is an influence of glycosylation on the transport mechanism and therefore not every PAG can be found in milk.

## Conclusions

In conclusion, a new Sandwich-ELISA was developed for the detection of boPAG in serum and milk of pregnant cattle within one system. This is time saving for farmers and more efficient for laboratories. From fourth week after insemination onwards, the Sandwich-ELISA was able to identify a cow as being pregnant with high sensitivity (97% in serum; 95% in milk) and specificity (96% in serum; 91% in milk). The detected boPAG-profile showed a typical pattern as described by other studies. With the possibility to measure boPAG concentration in whole milk, stressful effects during sampling (e.g. of venepuncture) are avoided and there is no need for special equipment or experience. Furthermore, to the best of our knowledge, only a few assays are described, which are able to quantify PAG in bovine milk and there is only one commercially available ELISA for detection of PAG in bovine milk which is designed as qualitative ELISA. The use of a quantitative assay has some advantages over a qualitative assay in research and clinical purposes. The quantification of an analyte gives more detailed information about the concentration range in different physiological states and over time (e.g. during pregnancy). Furthermore, it allows the comparison of concentrations between different individuals. This is not possible with qualitative or semi quantitative results.

For the reasons mentioned above, the described quantitative Sandwich-ELISA could be a very useful tool for pregnancy diagnosis in cattle.

## Supporting information

S1 Fig Comparison of concentrations from 37 serum samples of pregnancy-associated glycoproteins between the new developed Sandwich-ELISA and the ELISA established by Friedrich and Holtz [9] after standardization. The correlation was estimated with a linear regression (y = 1.017x, R^2^ = 0.91, *P*<0.001).
(TIF)

S2 Fig ROC curve to determine the optimal cutoff value for serum pregnancy testing. The Youden’s index was used to find the best cutoff value that optimizes sensitivity and specificity (indicated by the red dot).
(TIF)

S3 Fig ROC curve to determine the optimal cutoff value for milk pregnancy testing. The Youden’s index was used to find the best cutoff value that optimizes sensitivity and specificity (indicated by the red dot).
(TIF)

S1 Table Confusion matrix for evaluation of sensitivity, specificity, positive predictive value, negative predictive value, and accuracy in serum at a threshold value of 0.4 ng/ml. (PDF)

S2 Table Confusion matrix for evaluation of sensitivity, specificity, positive predictive value, negative predictive value, and accuracy in serum at a threshold value of 1.0 ng/ml. (PDF)

S3 Table Confusion matrix for evaluation of sensitivity, specificity, positive predictive value, negative predictive value, and accuracy in serum at a threshold value of 1.5 ng/ml. (PDF)

S4 Table Confusion matrix for evaluation of sensitivity, specificity, positive predictive value, negative predictive value, and accuracy in serum at a threshold value of 2.0 ng/ml. (PDF)

S5 Table Confusion matrix for evaluation of sensitivity, specificity, positive predictive value, negative predictive value, and accuracy in serum at a threshold value of 2.5 ng/ml. (PDF)

S6 Table Confusion matrix for evaluation of sensitivity, specificity, positive predictive value, negative predictive value, and accuracy in milk at a threshold value of 0.01 ng/ml. (PDF)

S7 Table Confusion matrix for evaluation of sensitivity, specificity, positive predictive value, negative predictive value, and accuracy in milk at a threshold value of 0.0165 ng/ml. (PDF)

S8 Table Confusion matrix for evaluation of sensitivity, specificity, positive predictive value, negative predictive value, and accuracy in milk at a threshold value of 0.02 ng/ml. (PDF)

S9 Table Confusion matrix for evaluation of sensitivity, specificity, positive predictive value, negative predictive value, and accuracy in milk at a threshold value of 0.025 ng/ml. (PDF)

S10 Table Confusion matrix for evaluation of sensitivity, specificity, positive predictive value, negative predictive value, and accuracy in milk at a threshold value of 0.16 ng/ml. (PDF)
