# Supplementary figures and images for: Establishment of a Sandwich-ELISA for simultaneous quantification of bovine pregnancy-associated glycoprotein in serum and milk
Source: PLoS One. 2021 May 12;16(5):e0251414. doi: 10.1371/journal.pone.0251414 (PMC8115853; doi:10.1371/journal.pone.0251414)

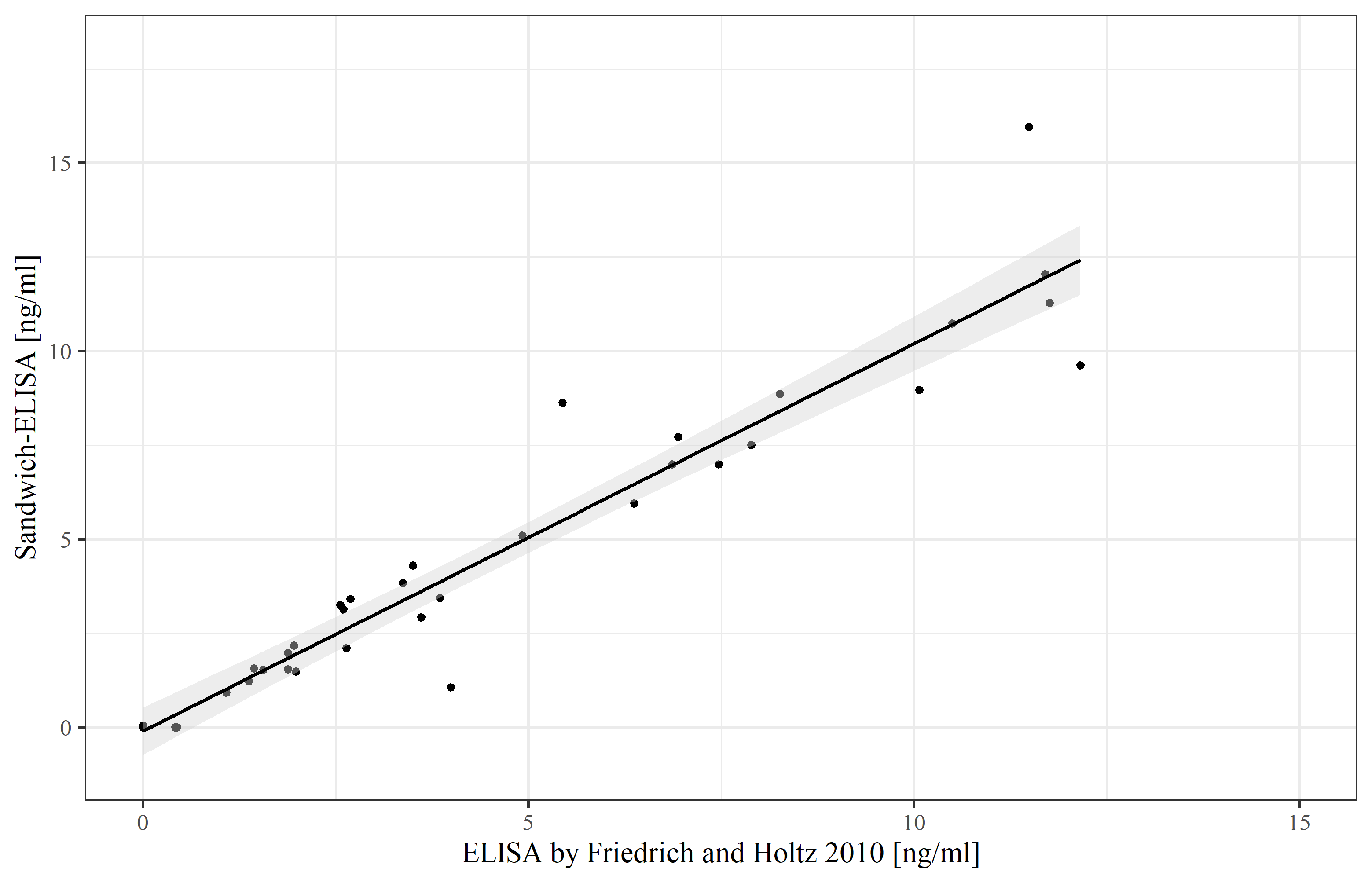

Supplement: S1 Fig — The correlation was estimated with a linear regression (y = 1.017x, R2 = 0.91, P<0.001). (TIF) [file pone.0251414.s001.tif]

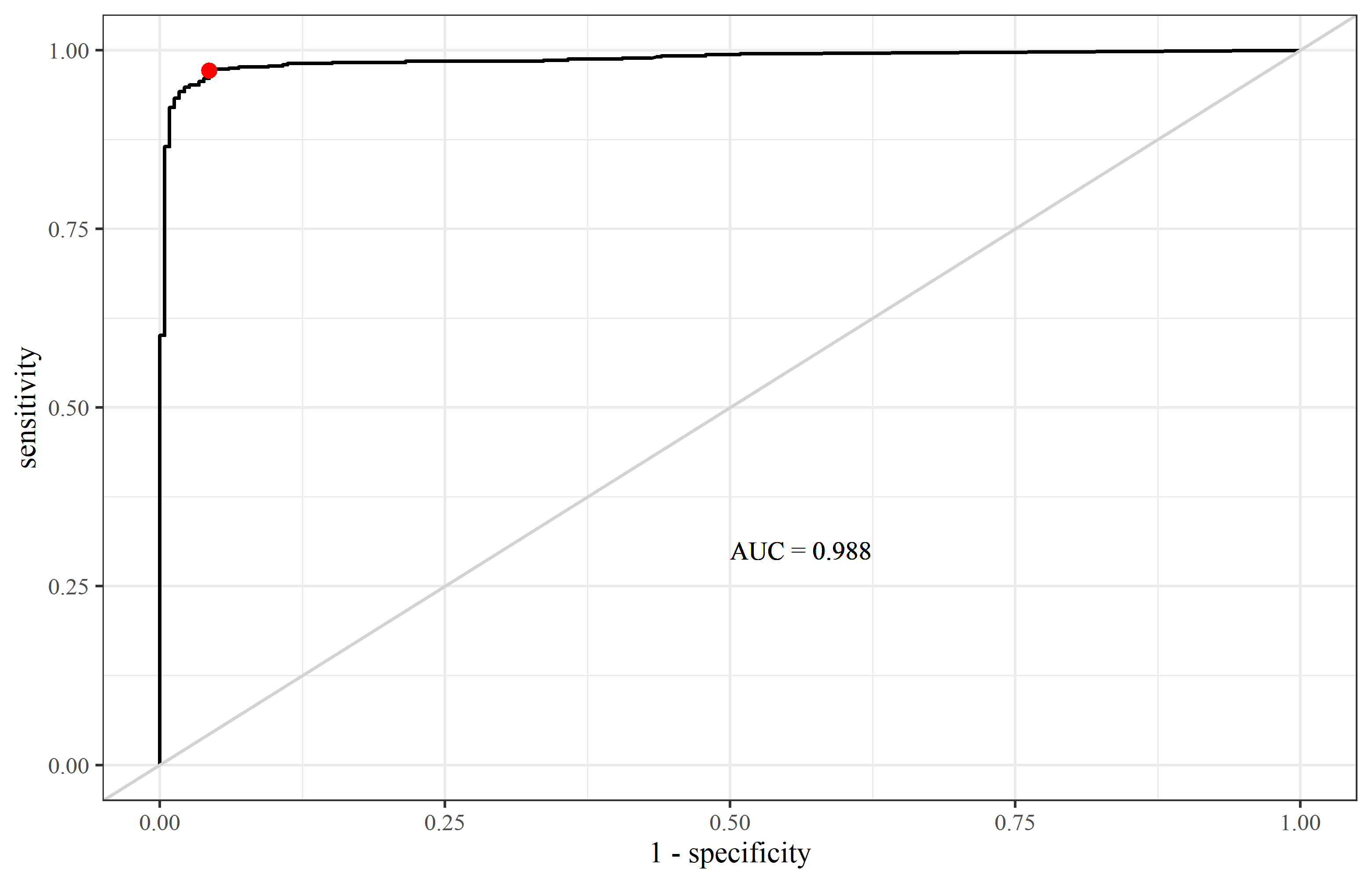

Supplement: S2 Fig — The Youden’s index was used to find the best cutoff value that optimizes sensitivity and specificity (indicated by the red dot). (TIF) [file pone.0251414.s002.tif]

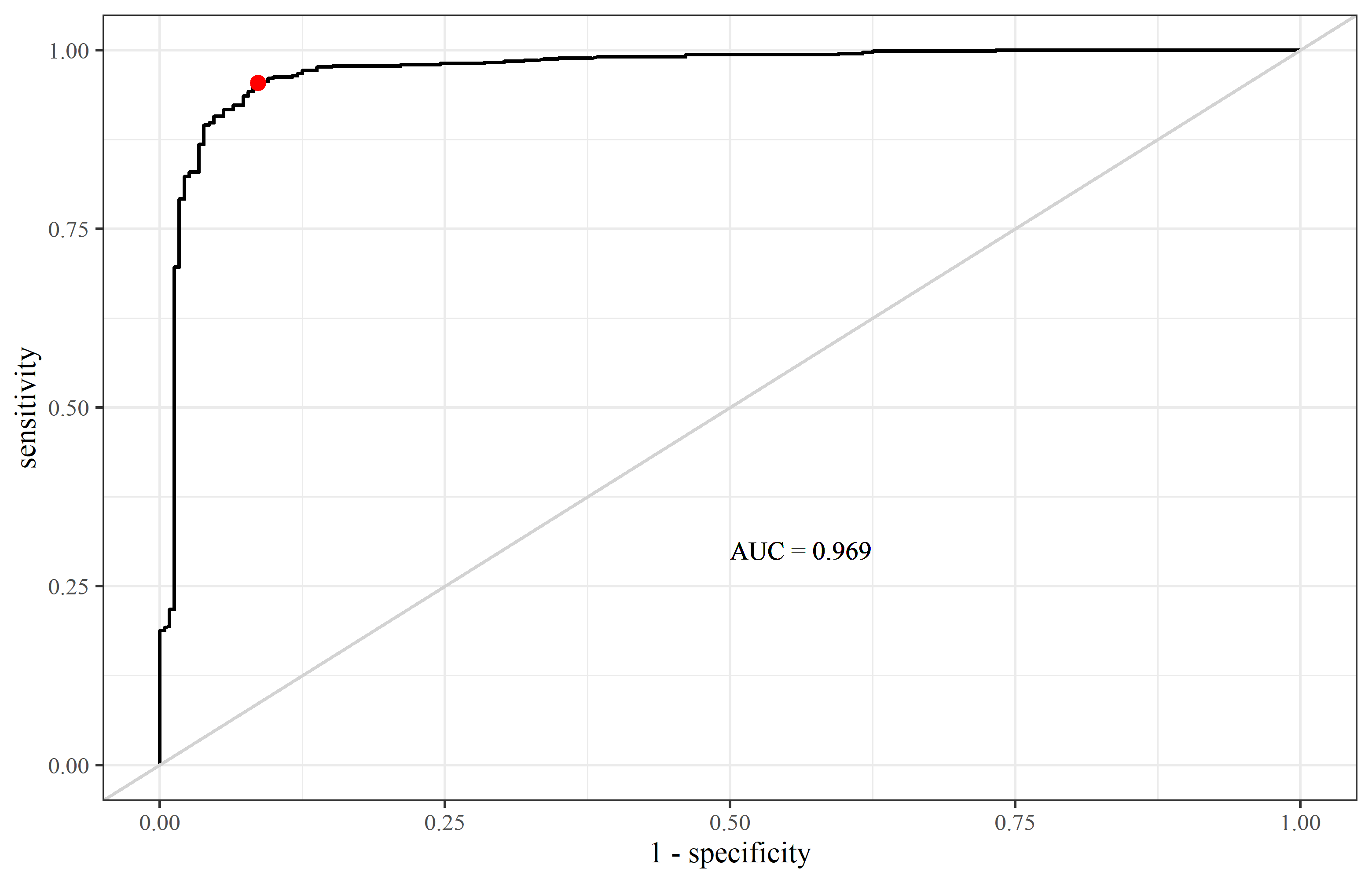

Supplement: S3 Fig — The Youden’s index was used to find the best cutoff value that optimizes sensitivity and specificity (indicated by the red dot). (TIF) [file pone.0251414.s003.tif]
