# Supplementary material for: Establishment of a Sandwich-ELISA for simultaneous quantification of bovine pregnancy-associated glycoprotein in serum and milk
Source: PLoS One. 2021 May 12;16(5):e0251414. doi: 10.1371/journal.pone.0251414 (PMC8115853; doi:10.1371/journal.pone.0251414)
Supplement: S3 Table — (PDF) [file pone.0251414.s006.pdf]

S3 Table. Confusion matrix for evaluation of sensitivity, specificity, positive predictive value, negative predictive value, and accuracy in serum at a threshold value of 1.5 ng/ml.

Threshold 1.5 ng/ml			
PAG-ELISA	Pregnant	Non-Pregnant	Total ∑
Pregnant	626	2	628
Non-Pregnant	40	153	193
Total ∑	666	155	821
