# Supplementary material for: Establishment of a Sandwich-ELISA for simultaneous quantification of bovine pregnancy-associated glycoprotein in serum and milk
Source: PLoS One. 2021 May 12;16(5):e0251414. doi: 10.1371/journal.pone.0251414 (PMC8115853; doi:10.1371/journal.pone.0251414)
Supplement: S5 Table — (PDF) [file pone.0251414.s008.pdf]

S5 Table. Confusion matrix for evaluation of sensitivity, specificity, positive predictive value, negative predictive value, and accuracy in serum at a threshold value of 2.5 ng/ml.

Threshold 2.5 ng/ml			
PAG-ELISA	Pregnant	Non-Pregnant	Total ∑
Pregnant	589	1	590
Non-Pregnant	77	154	231
Total ∑	666	155	821
