# Supplementary material for: Establishment of a Sandwich-ELISA for simultaneous quantification of bovine pregnancy-associated glycoprotein in serum and milk
Source: PLoS One. 2021 May 12;16(5):e0251414. doi: 10.1371/journal.pone.0251414 (PMC8115853; doi:10.1371/journal.pone.0251414)
Supplement: S7 Table — (PDF) [file pone.0251414.s010.pdf]

S7 Table. Confusion matrix for evaluation of sensitivity, specificity, positive predictive value, negative predictive value, and accuracy in milk at a threshold value of 0.0165 ng/ml.

Threshold 0.0165 ng/ml			
PAG-ELISA	Pregnant	Non-Pregnant	Total ∑
Pregnant	603	12	615
Non-Pregnant	30	130	160
Total ∑	633	142	775
